# Dynamic Behavior of p53 Driven by Delay and a Microrna-34a-Mediated Feedback Loop

**DOI:** 10.3390/ijms21041271

**Published:** 2020-02-13

**Authors:** Chunyan Gao, Haihong Liu, Fang Yan

**Affiliations:** 1 Department of Mathematics, Yunnan Normal University, Kunming 650092, China; 2 Department of Dynamics and Control, Beihang University, Beijing 100191, China

**Keywords:** p53-Mdm2 pathway, time delay, miR-34a, feedback loops

## Abstract

The tumor suppressor protein p53 is a critical hub in the comprehensive transcriptional network that inhibits the growth of cells after acute stress stimulation. In this paper, an integrated model of the p53 signaling pathway in response to DNA damage is proposed and the p53 stability and oscillatory dynamics are analyzed. Through theoretical analysis and numerical simulation, we find that the delay as a bifurcation parameter can drive the p53-Mdm2 module to undergo a supercritical Hopf bifurcation, thereby producing oscillation behavior. Moreover, we demonstrate how the positive feedback loop formed by p53* and microRNA-34a (miR-34a) with the feature of double-negative regulation produces limit-cycle oscillations. Further, we find that miR-34a can affect the critical value of Hopf bifurcation in delay-induced p53 networks. In addition, we show that ATM, once activated by DNA damage, makes p53* undergo two Hopf bifurcations. These results revealed that both time delay and miR-34a can have tumor suppressing roles by promoting p53 oscillation or high level expression, which will provide a perspective for promoting the development of anti-cancer drugs by targeting miR-34a and time delay.

## 1. Introduction

The importance of p53 in preventing tumor formation is indicated by mutations of the p53 gene found in more than half of human cancers [1,2]. Tumor suppressor p53 is located at a junction of the cellular signaling network and can be activated by DNA damage, cellular stress and improper mitogenic stimulation [3,4]. P53 integrates these signals and mainly acts as a transcription factor to regulate the expression of downstream target genes, and thereby induces different cellular outcomes such as cell cycle arrest and apoptosis [5,6]. The former promotes cell repair and survival, while the latter provides an effective way to remove irreparable damaged cells and avoid transmitting damage to the next generation of cells. Previously, it was proposed that p53 protein dynamics were of vital importance to cellular fate decisions in response to DNA damage, which also emphasizes the crucial role of p53 oscillation in cancer cell decision making [7].

Negative feedback has the potential to generate limit-cycle oscillations and is considered as a necessary structure for biochemical oscillators [8]. Indeed, Mdm2 protein is p53-inducible and functions as a negative regulator of p53, thereby confirming a negative feedback which is fundamental for p53 oscillations [9]. However, in this process wherein p53 acts as a transcription factor to induce Mdm2 gene expression, there are inevitable time delays which often result in complex dynamical behavior [10,11,12,13]. For example, Monk [10] pointed out that time delays drive p53 oscillation in a simple negative feedback system. After that, Ma, et al. [11] proposed that the negative feedback loop formed by p53 and Mdm2 with a long time delay could produce coordinated oscillatory dynamics upon IR stimulation in single cells. Therefore, the effect of delay on p53 oscillation cannot be neglected.

MicroRNAs (miRNAs) are a class of noncoding RNAs of approximately 18–25 nucleotides in length that are involved in post-transcriptional regulation of gene expression [14]. It has been reported that aberrant expression of miRNAs and a global decrease of their levels are often observed in multiple human cancer types [15,16]. For example, a class of microRNAs, microRNA-34a (miR-34a), has been found in experiments to have a low expression level in unfavorable primary neuroblastoma (NB) tumors and cell lines, and the cell proliferation is significantly reduced when the microRNA is re-introduced into NB cell lines. It suggested that miR-34a functions as a potential tumor suppressor by inducing apoptosis in NB cells [17]. Intriguingly, a set of studies revealed that miR-34a forms a positive feedback loop with p53 [18,19,20,21]. Tt has been reported that robust oscillations can be accomplished through a combination of negative and positive feedback loops rather than a single negative feedback loop [22,23]. Therefore, the positive feedback loop mediated by miR-34a should integrated in to the p53 network.

Based on the above biological fact, in this paper, a mathematical model of with seven major components and a critical time delay is proposed to investigate how cell cycle progression and cell fate decisions are well coordinated by transcription factors p53 and miR-34a in response to DNA damage. On the one hand, based on the stability theory of delayed differential equation, the stability and oscillation of the proposed model with and without time delay were investigated. They revealed that the time delay required for the synthesis process of Mdm2 gene expression is vitally important for sustained p53 oscillations. On the other hand, according to the bifurcation theory, the effect of positive feedback loops formed by miR-34a and p53 was explored. That showed that the positive feedback loop mediated by miR-34a can control stress-induced p53 oscillation. These findings are well consistent with the existing biological phenomena [11,13,24,25]. Moreover, we also predicted that the p53 oscillatory behavior is more sensitive than that without miR-34a and the critical value of Hopf bifurcation of p53 networks miR-34a can be affected by miR-34a. These results may provide clues for developing drugs to target miR-34a and p53 for cancer treatment.

## 2. Mathematical Model

**Figure 1:**
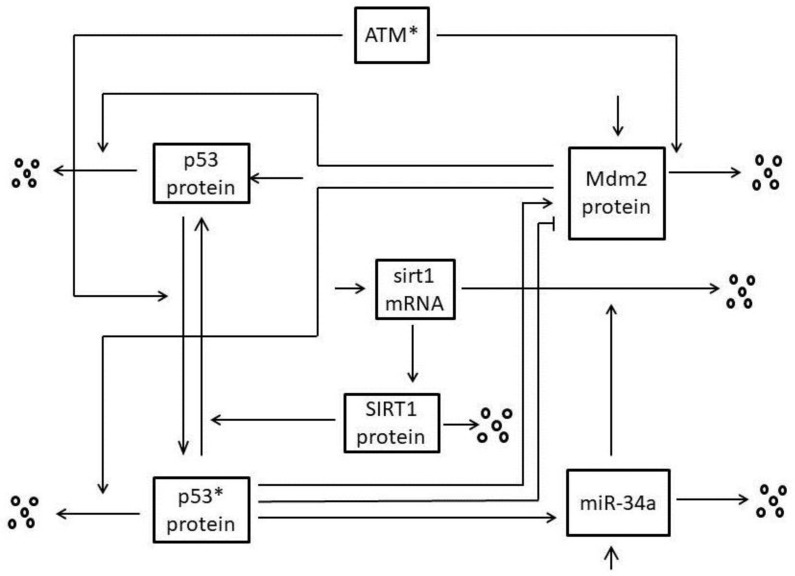
The p53 signal network. ATM* relays the DNA damage signal and induces activation of p53 from the inactive state to the active state (p53*). P53* is a transcriptional factor that can effectively promote the production of Mdm2; during that process there are inevitably time delays. Here, the time delay covers the time needed during both Mdm2 transcription and translation, which is recorded as τ. In turn, Mdm2 can bind to p53 and p53*, resulting in enhanced degradation of p53 and p53*. On the other hand, p53* can also activate miR-34a. Subsequently, miR-34a inhibits SIRT1 by promoting the degradation of sirt1 mRNA, which leads to an increase of acetylated p53. In addition, Mdm2 protein is degraded by a mechanism stimulated by ATM*.

According to the biochemical reactions among p53, p53 * protein, Mdm2 protein, miR-34a, sirt1-miR-34a, sirt1 mRNA and SIRT1 protein, the mathematical model can be directly construed from Figure 1 as follows:


$$\frac{dx_{1}\left(t\right)}{dt}=k_{sp53}+k_{da1}x_{7}\left(t\right)\frac{x_{2}\left(t\right)}{x_{2}\left(t\right)+k_{0}}-k_{dp531}x_{1}\left(t\right)+k_{rp}x_{2}\left(t\right)-k_{dp532}x_{3}\left(t\right)\frac{x_{1}\left(t\right)}{x_{1}\left(t\right)+k_{d1}}-k_{fp}A\frac{x_{1}\left(t\right)}{x_{1}\left(t\right)+k_{p}},$$

$$\frac{dx_{2}\left(t\right)}{dt}=k_{fp}A\frac{x_{1}\left(t\right)}{x_{1}\left(t\right)+k_{p}}-k_{rp}x_{2}\left(t\right)-k_{dp53s}x_{3}\left(t\right)\frac{x_{2}\left(t\right)}{x_{2}\left(t\right)+k_{d2}}-k_{da1}x_{7}\left(t\right)\frac{x_{2}\left(t\right)}{x_{2}\left(t\right)+k_{0}},$$

$$\frac{dx_{3}\left(t\right)}{dt}=k_{smd1}+k_{smd2}\frac{x_{2}{\left(t-\tau \right)}^{4}}{x_{2}{\left(t-\tau \right)}^{4}+k^{4}}-k_{dmdm20}x_{3}\left(t\right)-k_{dmdm21}\frac{A}{A+j_{atm}}x_{3}\left(t\right),$$

$$\frac{dx_{4}\left(t\right)}{dt}=k_{smi1}+k_{smi2}\frac{x_{2}{\left(t\right)}^{4}}{x_{2}{\left(t\right)}^{4}+j_{1}{}^{4}}-k_{dm1}x_{4}\left(t\right)-k_{on2}x_{6}\left(t\right)x_{4}\left(t\right)+k_{off2}x_{5}\left(t\right),$$

$$\frac{dx_{5}\left(t\right)}{dt}=k_{on2}x_{6}\left(t\right)x_{4}\left(t\right)-k_{off2}x_{5}\left(t\right)-k_{smir}x_{5}\left(t\right),$$

$$\frac{dx_{6}\left(t\right)}{dt}=k_{ss}-k_{ssi}x_{6}\left(t\right)-k_{on2}x_{6}\left(t\right)x_{4}\left(t\right)+k_{off2}x_{5}\left(t\right),$$

$$\frac{dx_{7}\left(t\right)}{dt}=r_{sirt1}x_{6}\left(t\right)-k_{sirt1}x_{7}\left(t\right).$$


**Figure 2:**
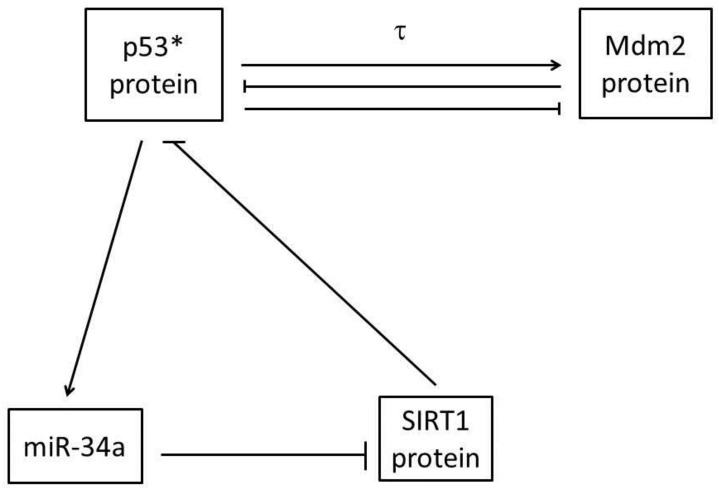
A simplified relationship containing the main components of the p53 signal network. The central node p53* connects two feedback loops. One is a negative feedback loop formed by p53* and Mdm2 with time delay, and the other is a positive feedback loop formed by p53* and miR-34a with the feature of double-negative regulation.

Here, *x_i_*(*t*) (*I* = 1,2,3,4,5,6,7) represent the concentrations at time t of inactive p53 protein, p53* protein, Mdm2 protein, miR-34a, sirt1-miR-34a, sirt1 mRNA and SIRT1 protein respectively. It is noteworthy that our model contains two feedback loops connected by the central node p53*, which is shown in the Figure 2. One is a negative feedback loop formed by p53* and Mdm2 with time delay, based on previously published work [11]; namely, the p53*-Mdm2 loop. This circuit alone can produce stable oscillations of p53 and Mdm2 in response to sufficiently strong ATM activation [11]. Specifically, as a sensitive and reliable detector of DNA damage [26,27], ATM* induces reaction of p53 from the inactive state to the active state (p53*) by post-translational modifications, such as phosphorylation, methylation and acetylation, on multiple different sites [28]. In particular, C-terminal acetylation of p53 on lysine 382 is indispensable to p53 activation and stability, which is shown in blue in Figure 1. As a transcription factor, p53* was able to promote the Mdm2 gene to synthesize the Mdm2 protein [29]. At the same time, phosphorylation of p53 on serine 15 negates MDM2, which is shown in red in Figure 1 and Figure 2 [29]. This process required certain time delays involving transcription, translation and transportation of Mdm2 molecules, denoted τ [12]. Normally, typical transcript elongation and processing rates would result in a time delay of around 15-20 min [30,31]. Similarly, synthesis of a typical protein from mRNA takes around 1-3 min and results in a translational delay [31]. Nuclear mRNA export has been estimated at 4 min [31]. Therefore, we estimate that the total delay time fluctuation ranges from 20 to 30 minutes. Subsequently, Mdm2 promotes the degradation of p53 and p53* protein [32].

The other is a positive feedback loop formed by p53* and miR-34a; namely, the p53*-miR-34a loop. Specifically, p53 acts as a transcription factor to increase expression of a set of miRNAs that includes miR-34a [33,34,35,36]. Subsequently, miR-34a regulates silencing of information regulator 1 (SIRT1) expression by inhibiting sirt1 mRNA translation [18,37]. In irradiated cells, SIRT1 proteins act as post-translational modification antagonists of p53, thereby inhibiting the transcriptional activity of p53 protein [38,39]. Therefore, the p53*-mir-34a loop is characterized by double negative regulation.

## 3. Results

Table 1 gives a complete list of model parameters and default values, and all the rate constants are used in all calculations, except where parameters are varied, or where noted otherwise. The numerical simulations were performed by Mathematica 10 and XPPAUT.

### 3.1. The Effect of Mir-34a on p53 Oscillations without Time Delay

**Figure 3:**
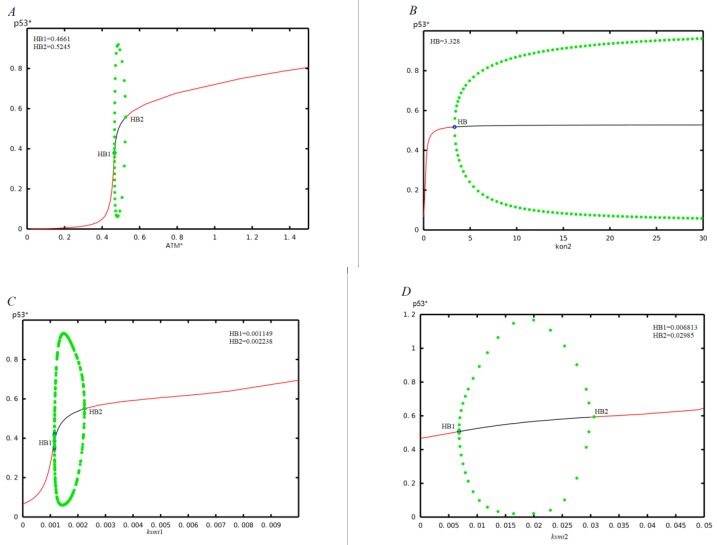
The bifurcation diagram of p53* concentration and model parameters. (**A**), (**B**), (**C**) and (**D**) correspond to the parameters ATM*, k_on2_, k_smi1_ and k_smi2_, respectively.

To further understand the effect of miR-34a in p53-Mdm2 networks, we performed bifurcation analysis as shown in Figure 3. The green dots represent the bounds of p53* oscillation amplitude at oscillation state (black line), and the red line represents stationary steady state.

Under normal circumstances, ATM is in an inactive state. When stimulated by DNA damage, ATM behaves as a Double Strand Breaks (DSB) sensor, which is activated by phosphorylation, and the phosphorylated ATM is denoted by ATM* [43]. Biological experiments have shown that ATM* can induce oscillatory expression of p53 [44]. We now study how ATM* levels affect p53 expression levels. For this purpose, we plotted the bifurcation diagram of the p53* concentration versus ATM* concentration, as shown in Figure 3A. At the low level of expression of the ATM*, p53* remains predominantly in the inactive forms, and the corresponding steady state is stable. As the ATM* increases, p53* exhibits a sustained oscillation, and then goes again to the stable, high-level state. As expected, these results indicate that the ATM* level can lead to two Hopf bifurcation points at HB1=0.4661 and HB2=0.5245, respectively, and only a certain ATM* level can drive p53 oscillations, which is in good agreement with the results reported in the existing studies [12].

Subsequently, we analyze the effect of miR-34a-mediated positive feedback loop, which can be reflected by the role of parameters related to miR-34a on the p53 oscillation. In particular, we probed the effects of three parameters (k_on2_, k_smi1_ and k_smi2_) on p53* oscillation without time delay (i.e., τ = 0). The larger these three parameters are, the stronger the strength of positive feedback mediated by miR-34a are.

First, we obtained the bifurcation diagrams of the p53* concentration versus the association rate between miR-34a and sirt1 mRNA (k_on2_), as shown in Figure 3B. It is shown that p53* is so sensitive to the association rate k_on2_ of the miR-34a and sirt1 mRNA, and the parameter value k_on2_ = 3.328 is sufficient to induce p53* to be oscillatory. Moreover, the amplitudes of these oscillations increase with increasing k_on2_.

Second, the bifurcation diagrams of the p53* concentration versus the basal induction rate of miR-34a (k_smi1_) is shown in Figure 3C. For k_smi1_ < 0.001149, p53* level remains in a low steady state owing to weak promotion of miR-34a, and slowly rises with increasing k_smi1_. Moreover, there exist two Hopf bifurcation points at HB1 and HB2, respectively. As k_smi1_ increases gradually, a Hopf bifurcation labeled as HB1 appears at k_smi1_ = 0.001149, beyond which p53* concentration undergoes periodical oscillations. When k_smi1_ is increased to the other Hopf bifurcation labeled as HB2 at k_smi1_ = 0.002238, oscillations vanish. Importantly, one can see that the amplitudes of these oscillations from HB1 to HB2 rise first and then decrease with increasing k_smi1_. Furthermore, when k_smi1_ > 0.002238, p53* remains at high levels. These results indicate that the threshold level of the input basal induction rate of miR-34a is a sufficient condition for generating p53* oscillations.

Third, we calculated the bifurcation diagrams of the p53* concentration versus the p53*-induced transcription rate of miR-34a (k_smi2_), as shown in Figure 3D. As the p53*-induced transcription rate of miR-34a goes from low values to higher values, the p53* level goes from a stable state into a series of sustained oscillations and then again to stable state. In other words, when k_smi2_ gradually increases from 0, there are two Hopf bifurcation points labeled HB1 and HB2 in turn. The first Hopf bifurcation point HB1 appears at the parameter k_smi2_ = 0.006813. When k_smi2_ continues to increase, the concentration of p53* will oscillate periodically. When k_smi2_ is increased to the other Hopf bifurcation labeled as HB2 at k_smi2_ = 0.02985, oscillations vanish. Therefore, a certain range of the p53*-induced transcription rate of miR-34a is required to generate the p53* oscillations.

In summary, as the intensity of inhibition of sirt1 by miR-34a and the production of miR-34a are increased, p53 will jump from a low steady state to a continuous oscillation state and even to a high steady state. This means that miR-34a has a role in promoting cell cycle arrest and apoptosis, and thus inhibits cancer development.

**Figure 4:**
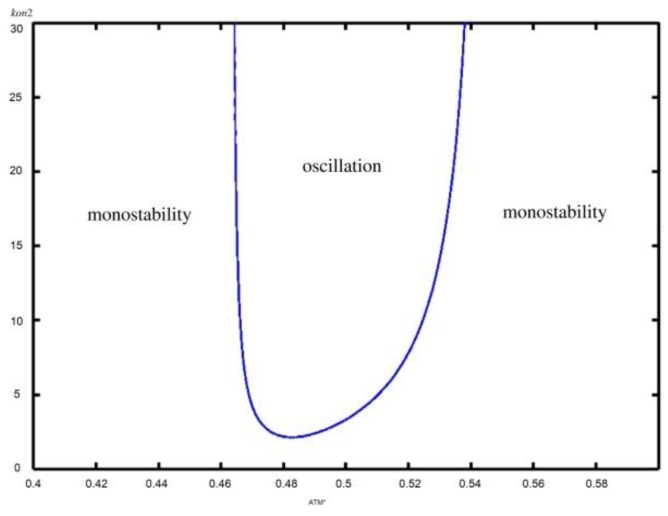
The bifurcation diagram of the k_on2_ versus ATM* concentration.

Additionally, we also plot the diagram of the k_on2_ versus ATM* concentration to further address the role of miR-34 on p53 oscillation, as shown in Figure 4. The blue line in the figure indicates the Hopf bifurcation point. Only when parameters k_on2_ and ATM* concentration are controlled in an appropriate range can p53* oscillate. Indeed, when the parameter k_on2_ is restricted to a specific value, with the increase of ATM* concentration, p53* enters the oscillating state from the monostable state, and then returns to the monostable state, which is consistent with Figure 3A. Similarly, when ATM* concentration is restricted to a specific medium value, p53* enters a sustained oscillation state from a monostable state with k_on2_ increases, which is consistent with Figure 3B. However, when the DNA damage is too small, that is, the ATM is small, even if the inhibition of sirt1 by miR-34a is enhanced, p53 does not oscillate. This is because the DNA damage is too small and does not require p53 oscillation to repair the damage. At the same time, when the DNA damage is too large, that is, the ATM is large, even if the inhibition of sirt1 by miR-34a is enhanced, p53 does not oscillate, but is at a high steady state. This indicates that the cells do not need to repair the damage by oscillation, but directly undergo apoptosis.

### 3.2. The Effect of Time Delay on the p53-mdm2 Network without MiR-34a

**Figure 5:**
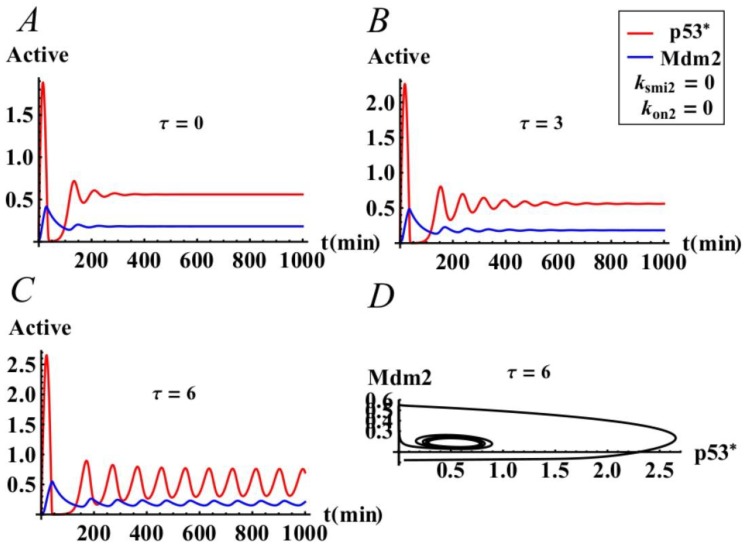
The effects of time delay τ on the p53-Mdm2 system of model (1) when there is no expression of miR34-a. (**A**) The positive equilibrium is asymptotically stable whenτ = 0. (**B**) The positive equilibrium is asymptotically stable when τ = 3 < τ_0_ = 5.1038. (**C**,**D**) The positive equilibrium is unstable when τ = 6 > τ_0_ = 5.1038.

p53 regulates the gene expression of Mdm2, which inevitably involves the transcription and translation time delay τ. In this section, we focus on the effect of time delay on p53 oscillation by numerical simulation. To this end, we do not consider the role of miR-34a on p53; i.e., parameters k_smi2_ = 0 and k_on2_ = 0. Figure 5 displays the time histories of the levels of p53* and Mdm2. Obviously, if there are no time delays in the system, the positive equilibrium is asymptotically stable, which can be seen in Figure 5A. Otherwise, if there exist time delays in the system, the effects of time delay on the p53-Mdm2 network are shown in Figure 5B–D. In particularly, when τ = 3 min, the system’s positive equilibrium point is asymptotically stable, and when τ = 6 min, the system’s positive equilibrium point is no longer stable but is in periodic oscillation. This shows that as the time delay gradually increases and exceeds the critical value, the positive equilibrium point loses its stability and becomes an oscillating state, which means that the system has experienced a Hopf bifurcation. The critical value of time delay for the Hopf bifurcation is calculated to be τ_0_ = 5.10388. Therefore, we find that the delay as a bifurcation parameter makes the p53-Mdm2 oscillator undergo a supercritical Hopf bifurcation without considering the miR-34a, which is consistent with the conclusions of some researchers [10,11].

### 3.3. Co-Regulation of the p53 Oscillatios by Time Delay and Mir-34a

**Figure 6:**
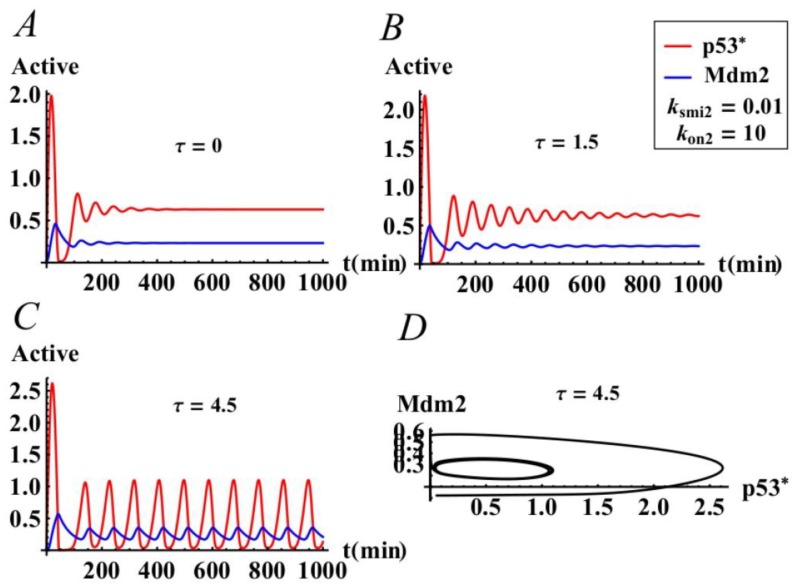
The effects of time delay τ on the p53-Mdm2 system of model (1) when there is certain expression of miR34-a. (**A**) The positive equilibrium is asymptotically stable when τ = 0. (**B**) The positive equilibrium is asymptotically stable when τ = 1.5 < τ_0_ = 3.5578. (**C**,**D**) The positive equilibrium is unstable when τ = 4.5 > τ_0_ = 3.5578.

In this part, we study how time delay and miR-34a co-regulate the p53 oscillations. As shown in Figure 6, we find that p53* still exhibits dynamic behavior similar to Figure 5 by introducing p53*-miR-34a into positive feedback loop. But the key difference is that the critical value of Hopf bifurcation appears earlier; i.e., τ_0_’=3.55785 < τ_0_ = 5.10388. This indicates that the p53* oscillatory behavior is more sensitive in the p53 network with the positive feedback loop induced by miR-34a, which suggests that miR-34a accelerates the response of p53 to DNA damage. Moreover, compared with a negative feedback loop with time delay, -sustained and robust P53 oscillation is achieved more easily by a coupled loop constructed by positive and negative feedback loops. In addition, it suggested that the positive feedback loop mediated by miR34-a can effectively accelerate the response of p53 to DNA damage so that it inhibits the occurrence of diseases caused by DNA damage. [45].

**Figure 7:**
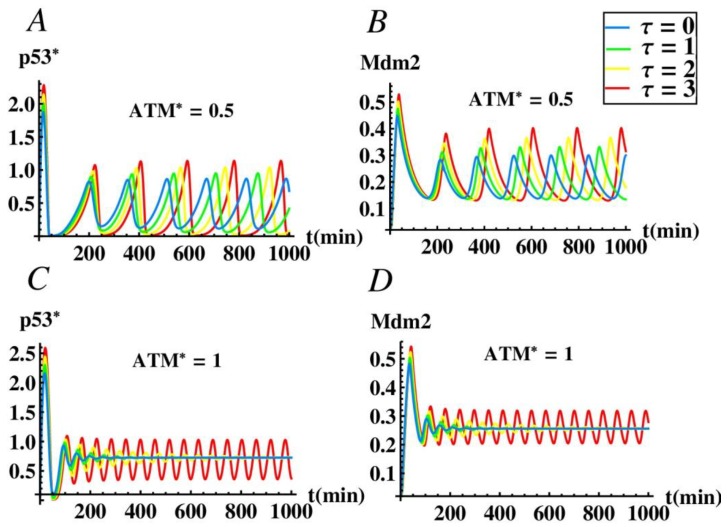
The effect of time delay on the dynamics of the p53-mdm2 system. (**A**) The time evolution process of the p53* concentration when ATM* = 0.5. (**B**) The time evolution process of the Mdm2 concentration when ATM* = 0.5. (**C**) The time evolution process of the p53* concentration when ATM* = 1. (**D**) The time evolution process of the Mdm2 concentration when ATM* = 1.

As mentioned in Section 3.1, miR-34a plays an important role in causing p53 oscillation without time delay. In order to further study the effect of time delay on the oscillation of p53 network, we also studied the influence of parameter τ on the amplitude and the period of the oscillation with the presence of miR-34a by numerical simulation. Figure 7A, B show that when A = 0.5, p53* and Mdm2 still exhibit oscillatory behavior without time delay, which is consistent with Figure 3A. At this time, although the change of time delay does not affect the stability of the system, we can see that the oscillation amplitude and period of the system increase with the increase of the delay. It is worth mentioning that in cells without damage, p53 remains at a low level [46,47]. However, when DNA is damaged, p53 accumulates, inducing genes to block DNA synthesis, repair damage or cause cell apoptosis [48,49]. Especially, p53 oscillation is beneficial to the repair of damaged cells, while the high level of p53 promotes the apoptosis of cells impossible to repair, or cancerous cells, avoiding inheritance [50]. Figure 7C,D shows that when A = 1, p53* and Mdm2 are stable state without delay, which is consistent with Figure 3A. As the delay τ increases and passes through the critical time delay τ_1_ = 2.7226, sustained oscillations of system can be observed. Moreover, both the amplitude and the period of the oscillations tend to increase with an increase of τ. Therefore, time delay plays a crucial role in the oscillation regulation of p53, and it is also known that miR-34a plays an indispensable role in the model.

## 4. Discussion

We have presented a mathematical model of the decision process with seven major components and a critical time delay. In our model, the dynamics of the p53-Mdm2 network were studied using the Hopf bifurcation theory and numerical simulations. From a biological point of view, under normal circumstances, the concentration of p53* is in a stable steady state and the concentration is low. When the cells meet mild or moderate DNA damage, p53* will show oscillating expression, which can lead to cell cycle arrest to repair DNA damage. When cells are stimulated by severe DNA damage, the concentration of p53* is stay at a stable steady state and the concentration is high, which promotes apoptosis. This means that as the degree of damage increases, p53* changes from a low stable steady state to an oscillating state and then to a high stable steady state. In this article, the degree of DNA damage is represented by the amount of ATM. Therefore, corresponding to the change of the concentration of p53* with ATM*, p53* experienced two Hopf bifurcations: the first time it changed from low steady state to oscillating state, and the second time it changed from oscillation state to high steady state. Similarly, we are also concerned about the effect of p53* on miR34-a, so the Hopf bifurcation of the concentration of p53* with the parameters kon2, ksmi1 and ksmi2 is discussed, which is shown in Figure 3. Considering the time delay as a bifurcation parameter, we studied the effect of time delay on p53* oscillation with or without miR-34a. It was found that time delay τ plays a key role in inducing Hopf bifurcation and in determining the amplitude and period of each oscillation. At the same time, we also found that the point of Hopf bifurcation induced by time delay was ahead of schedule after introducing miR-34a, which shows that miR-34a is also an indispensable factor affecting p53* oscillation. Then, in order to further study the role of miR-34a in our model, we performed a bifurcation analysis and found that the model parameters k_on2_, k_smi1_ and k_smi2_ could induce p53* oscillation in an appropriate range. Concretely, the results can be list as follows. First, consistent with the existing research results, we demonstrated that an association rate between miR-34a and sirt1 mRNA (k_on2_,) can drive p53* oscillations and the amplitude of the oscillation increases as its concentration increases. Second, we have shown that p53* undergoes the initial low-level steady state to the intermediate sustained oscillation state, and at the end reaches to the high-level steady state with the increase of the basal induction rate of miR-34a (k_smi1_). Finally, as a transcription factor, p53 promotes the production of miR-34a, and its transcription rate (k_smi2_) can induce the oscillation of p53* in an appropriate range. As expected, these results indicated that the positive feedback loop mediated by miR-34a could control stress-induced p53* oscillation. In addition, our results also indicate that a certain amount of miR-34a can also induce higher expression of p53*. Experiments have proven that when p53* was at a high level, the apoptosis program will be initiated, avoiding inheritance [51,52,53]. This suggests that miR-34a may be used as a potential anti-cancer tool. In conclusion, our research may provide a novel perspective for cancer treatment.

Although a new theoretical model is proposed in this paper to predict the dynamic behavior of p53 depending on the time delay and miR-34a, in fact the network is far from simple and involves some other feedback loops. For example, the double negative feedback loop miR34a-| MDM4 -| p53* and the bigger positive feedback loop miR34a-| MDM4 -> MDM2 -| p53*. These feedback loops undoubtedly increase the complexity of the network, so there may be more complex or new dynamic phenomena, which deserve further discussion and will be the next research topic we need to consider.

**Figure 8:**
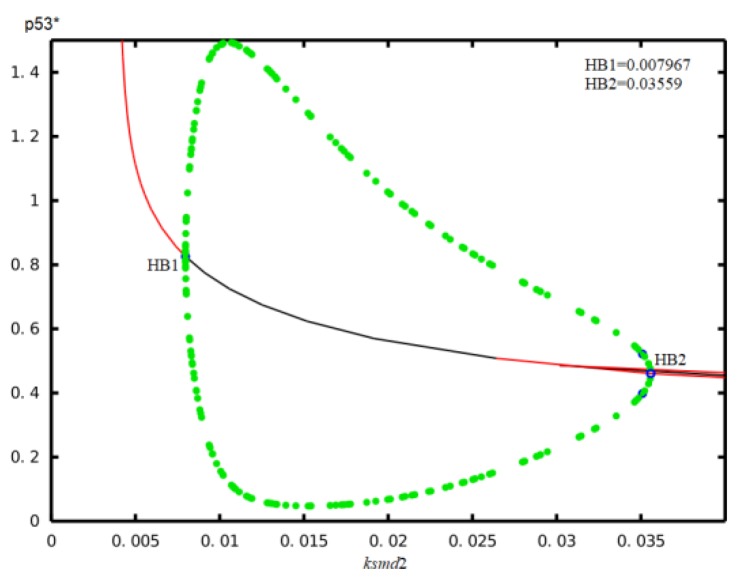
The bifurcation diagram of p53* concentration corresponds to the parameter k_smd2_.

**Figure 9:**
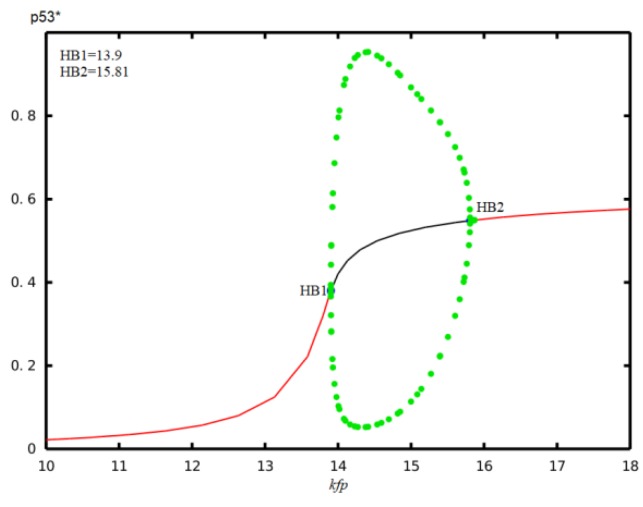
The bifurcation diagram of p53* concentration corresponds to the parameter k_fp_.

In our article, the main concern is the effect of other two factors, time delay and miR-34a, on the dynamic of activation of p53 to deduce the role of time delay and miR-34a on the p53-Mdm2 cancer network. Here, the different ways of activating p53 via different post-translational modifications are not handled differently, but we adopt the generally accepted biological fact that there is negative feedback regulation between p53 and Mdm2. For example, the effect of phosphorylation of p53 on serine 15 that negates MDM2 is reflected in the bifurcation diagram of p53* with respect to k_smd2_ shown in Figure 8. For k_smd2_ < 0.008, p53* level remains in a high steady state, and slowly declines with the increase of ksmd2. Once k_smd2_ is greater than 0.008 and less than 0.036, p53* concentration undergoes periodical oscillations. When k_smd2_ > 0.036, the oscillations of p53* vanished and the expression level stays at the low steady state. This bifurcation diagram suggested that the expression level of p53* is tightly depend on p53-dependent synthetic rate of Mdm2. Concretely, when the p53-dependent synthetic rate of Mdm2 is low, the p53* lies at a high steady state, which corresponds to cells with severe DNA damage, and the high level of p53* will promote cell apoptosis. When the p53-dependent synthetic rate of Mdm2 is low, the p53* is increased to a median range. The p53* then exhibits sustained oscillations, which correspond to cells with moderate DNA damage, and the oscillation of p53* will promote cell arrest. When the p53-dependent synthetic rate of Mdm2 was low, p53* was plentiful enough, so p53* switched to a low steady state, which corresponds to that of the normal cells. Moreover, C-terminal acetylation of p53 on lysine 382 is indispensable to p53 activation and stability, which is considered in a combined manner with other post-translational modification methods. The integrated effect of p53* on Mdm2 is reflected by the bifurcation diagram of p53* on p53-dependent synthetic rate of Mdm2, which is displayed in Figure 9. For k_fp_ < 13.9, p53* level remains in a low steady state, and slowly rises with increasing of k_fp_. Once k_fp_ is greater than 13.9 and less than 15.77, p53* concentration undergoes periodical oscillations. When k_fp_ > 15.77, the oscillations of p53* vanished and the expression level stayed at the high steady state. This bifurcation diagram suggested that the expression level of p53* is tightly depend on the activation rate regulated by post-translational modifications. Concretely, when the activation rate regulated by post-translational modifications is low, p53* will lie at a low steady state, which corresponds to that of normal cells. When the activation rate regulated by post-translational modifications is increased to a median range, p53* exhibits sustained oscillations, which corresponds to cells with moderate DNA damage, and the oscillation of p53* will promote cell arrest. When the activation rate regulated by post-translational modifications was big enough, p53* switched to a high steady state, which corresponds to cells with severe DNA damage, and the high level of p53* will promote cell apoptosis.

In addition, p53 can be activated at multiple different sites through post-translational modifications, for example, phosphorylation, methylation and acetylation, to regulate different downstream target genes. We believe that separately and comprehensively comparing and summarizing the differences of the activation of p53 and the corresponding downstream tumor suppressor genes caused by different post-translational modifications of p53 on multiple sites will help biologists to flexibly and purposefully regulate the p53 cancer network so as to effectively treat cancer. This is a very interesting topic, which may be the theme of our next research effort.

## 5. Materials and Methods 

In this paper, we systematically study the dynamical behavior of the p53-mdm2 system with a combination of theoretical predictions and numerical simulations. Particular attention is paid to the oscillatory behavior generated by Hopf bifurcation. From the perspective of mathematics and physics, bifurcation refers to the qualitative behavior of the system changes as the parameters change. In particular, Hopf bifurcation means that the system changes from a stable steady state to an oscillating state or from an oscillation state to a stable steady state as the parameters increase, which is often used to design biological and physical oscillations. In practice, stable steady state and oscillating state may correspond to different physiological states or biological functions. Therefore, it is extremely important at what rate the switching between these two states occurs. Using mathematical and physical methods to predict this switching point is of great significance for the control of certain biological issues. In addition, protein synthesis time delay in nonlinear dynamic systems often leads to rich dynamic behaviors, such as Hopf bifurcation, chaos and periodic oscillations. Here, we focus on the Hopf bifurcation caused by protein synthesis time delay and the positive feedback loop mediated by miR-34a. One of the conditions for satisfying the Hopf bifurcation theory is that the derivative of the real part to the parameter is greater than zero. Moreover, all of these bifurcation diagrams are drawn by using the software XPPAUT, which is a free crossing platform of dynamic system numerical simulation and bifurcation analysis. It is developed by Professor Eard Ermentrout at the Institute of Mathematical Sciences of the University of Pittsburgh, Pennsylvania. Its homepage is http://www.math.pitt.edu/~bard/xpp/xpp.html, where one can find more details. Other numerical simulations are plotted by mathematica10, which is a scientific computing software. It is founded by Stephen Wolfram in 1987. Its company is Wolfram Research, whose Corporate Headquarters is at Champaign, United States. On the grounds of biochemical reactions, the dynamic characteristics of p53 activity are analyzed and predicted based on a set of empirical data given in the published literature. If the empirical data of p53 activity have changed, the functional relationship in the mathematical model has not changed, but the parameters have changed, which can be analyzed by using the same method.

## Figures and Tables

**Table 1:** Parameters values used in numerical simulations.

Parameter	Description	Value	Reference
k_sp53_	Production rate of p53	0.2	[40]
k_da1_	Deactivation rate of p53* by SIRT1	3.3	[41]
k_0_	Michaelis constant of SIRT1-dependent p53 deactivation	0.0087	[41]
k_dp531_	Basal degradation rate of p53	0.02	[11]
k_dp532_	Mdm2-dependent degradation rate of p53	0.7	[40]
k_d1_	Threshold conc. for Mdm2-dependent p53 degradation	0.03	[11]
k_d2_	Threshold conc. for Mdm2-dependent p53* degradation	0.3	[11]
k_fp_	The post-translational modifications rate including phosphorylation, methylation, acetylation, etc. on multiple different sites of p53	15	[41]
kp	Michaelis constant of ATM*-dependent p53 phosphorylation	0.87	[41]
A	ATM* concentration	0~10	[12]
k_rp_	Dephosphorylation rate of p53*	0.2	[11]
k_dp53s_	Mdm2-dependent degradation rate of p53*	0.14	[40]
k_smd1_	Basal production rate of Mdm2	0.002	[41]
k_smd2_	P53-dependent synthetic rate of Mdm2	0.024	[41]
k	Michaelis constant of p53-dependent Mdm2 production	1	[11]
k_dmdm20_	Basal degradation rate of Mdm2	0.003	[40]
k_dmdm21_	ATM*-dependent degradation rate of Mdm2	0.05	[40]
k_smi1_	Basal induction rate of miR-34a	0.0018	[39]
k_smi2_	P53*-induced transcription rate of miR-34a	0.01	[39]
j_1_	Michaelis constant of p53*-dependent miR-34a transcription	1	Estimate
k_dmi_	Degradation rate of miR-34a	0.0078	[41]
k_on2_	Association rate between sirt1 mRNA and miR-34a	10	[41]
k_off2_	Dissociation rate of sirt1-miR-34a complex	0.13	[41]
k_smir_	Degradation rate of sirt1-miR-34a complex	0.062	[41]
k_ss_	Basal induction rate of sirt1 mRNA	0.01	[42]
k_ssi_	Degradation rate of sirt1 mRNA	0.062	[41]
r_sirt1_	Translation rate of SIRT1 protein	0.42	[41]
k_sirt1_	Degradation rate of SIRT1 protein	0.03	[41]
τ	Transcriptional and translation time delays	0∼30min	Estimate
